# Eosinophilic angiocentric fibrosis of the sinonasal tract

**DOI:** 10.1259/bjrcr.20150419

**Published:** 2016-05-05

**Authors:** Chunzi Jenny Jin, Bayardo Perez-Ordonez, Ian Witterick

**Affiliations:** ^1^ Department of Oncology, Cancer Centre of Southeastern Ontario at Kingston General Hospital, Queen’s University, Kingston, Canada; ^2^ Department of Pathology, University Health Network, University of Toronto, Toronto, Canada; ^3^ Department of Otolaryngology–Head and Neck Surgery, University of Toronto, Toronto, Canada

## Abstract

Eosinophilic angiocentric fibrosis (EAF) is an exceedingly rare and potentially disfiguring and obstructing benign lesion involving the upper airways. We report two cases of EAF originating from the nasal cavity in a 31-year-old female and a 58-year-old male exhibiting nasal obstructive symptoms, with imaging features and histopathology characteristic of EAF. Surgical excision was performed on one patient with a disfiguring nasal mass at a tertiary referral rhinology practice within a university centre. Summarized are the relevant clinical issues to increase awareness of this disease. The slow progression and rarity of the disease has previously resulted in diagnostic difficulty. We review the limited current literature surrounding the clinical features and treatment options for this progressive and potentially morbid condition. These cases reinforce that, while rare, inflammatory and fibrosing lesions in general should still be considered as part of the differential diagnosis in patients presenting with obstructive lesions in the sinonasal tract.

## Summary

Eosinophilic angiocentric fibrosis (EAF) is an exceedingly rare and potentially disfiguring and obstructing benign lesion involving the upper airways. We report two cases of EAF originating from the nasal cavity in a 31-year-old female and a 58-year-old male exhibiting nasal obstructive symptoms, with imaging features and histopathology characteristic of EAF. Surgical excision was performed on one patient with a disfiguring nasal mass at a tertiary referral rhinology practice within a university centre. Summarized are the relevant clinical issues to increase awareness of this disease. The slow progression and rarity of the disease has previously resulted in diagnostic difficulty. We review the limited current literature surrounding the clinical features and treatment options for this progressive and potentially morbid condition. These cases reinforce that, while rare, inflammatory and fibrosing lesions in general should still be considered as part of the differential diagnosis in patients presenting with obstructive lesions in the sinonasal tract.

## Case presentations

### Patient 1

**Figure 1.**
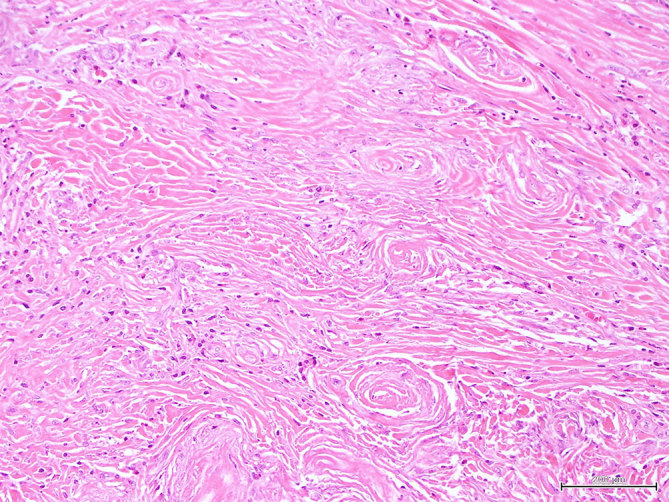
Patient 1. Nasal mass biopsy. Thick collagen bundles with perivascular fibrosis in an “onion-skin” type whorling pattern. Eosinophil-rich inflammatory infiltrate is also present (haematoxylin and eosin).

A 31-year-old female of Indian descent originally presented with chronic right-sided nasal obstruction, congestion and sinusitis. She previously underwent septoplasty and endoscopic sinus surgery 8 years ago. Her symptoms persisted and the deformity of her nasal bridge became more splayed, taking on the appearance of an external nasal mass. A biopsy revealed rich inflammatory infiltrate surrounding the blood vessels with a prominent onion-skin pattern, consistent with EAF extension into bony and skeletal muscle tissue (Figure 1).

A tertiary referral was then made to our department 3 years ago. Her main complaints were severe nasal blockage and sinus pain, but no epistaxis. The patient was otherwise in good health with no signs of systemic vasculitis or autoimmune disorder and she was not on any medication. There was no significant smoking or alcohol history. She had an allergy to penicillin. The patient had immigrated from India at the age of 16 years.

Physical examination revealed involvement of the nasal bones and cartilage, resulting in an enlargement and expansion of the nose. There was significant right-sided nasal passage obstruction with a soft tissue mass that had an oedematous appearance.

**Figure 2.**
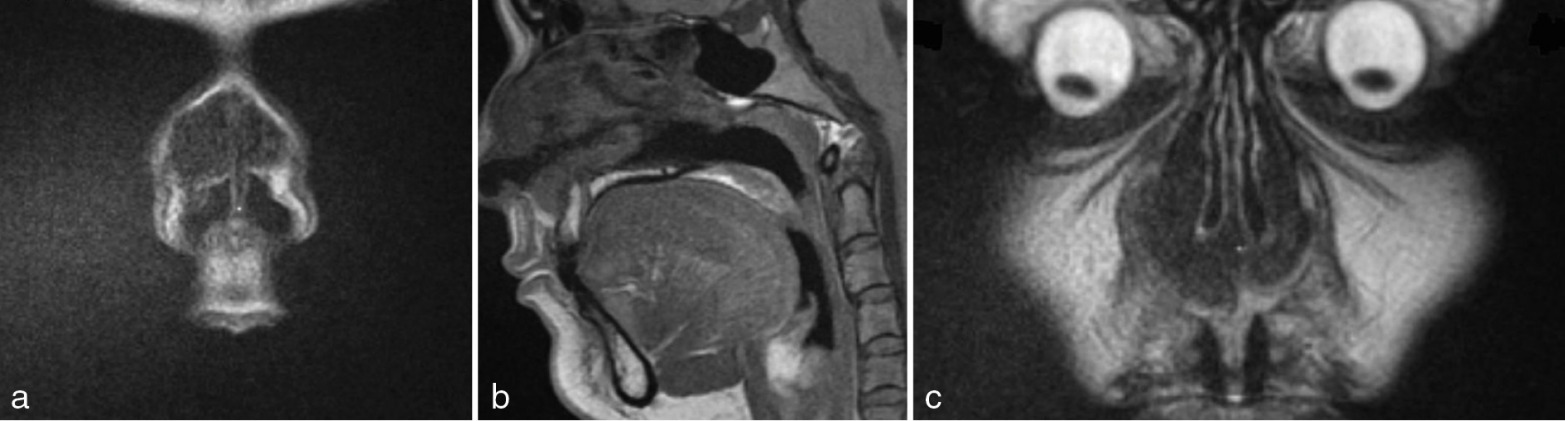
MRI of patient 1. (a) Coronal *T*_2_ (b) sagittal *T*_1_ and (c) coronal *T*_1_ weighted images showing a central soft tissue mass bilaterally infiltrating the nasolabial folds, inferior and middle turbinates, and nasal septum, with soft tissue thickening extending to the nasal bridge. There is mild narrowing of both nasal vestibules.

MRI showed a lobulated diffuse soft tissue mass of the nasolabial folds bilaterally, extending posteriorly to involve the anterior half of the inferior and middle turbinates as well as the anteroinferior nasal septum, mildly narrowing both nasal vestibules (Figure 2). There was soft tissue thickening extending to the nasal bridge.

Given the functional and cosmetic difficulties of gross total removal, the patient underwent subtotal resection, including removal of the nasal bones through an open septorhinoplasty approach. The resultant defect was reconstructed with rib grafts. The post-operative recovery was uneventful and the patient’s symptoms had improved. The pathology report confirmed EAF. 4 months postoperatively, the follow-up MRI documented residual disease along the right nasofacial groove with mild mass effect. Subsequent MRIs over 2 years of follow-up showed minimal progression. The patient continued to complain of mild but stable right nasal obstruction. A radiation oncology opinion was obtained and the patient was presented at the institutional tumour board. The consensus of opinion was that the risks associated with additional surgery, radiation or immunosuppressive therapy outweighed the potential benefits. In the absence of severe symptoms and no evidence of disease progression, the recommendation was for continued observation. The patient is being monitored and further treatment will be reconsidered should there be progression.

### Patient 2

A 58-year-old male presented with a palpable mass in the anterior nasal septum that had been gradually increasing in size over 1 year. His symptoms included bilateral nasal obstruction and persistent rhinorrhoea. There was no history of epistaxis, significant medical issues, medications or allergies. He had an 18 pack-year smoking history.

**Figure 3.**
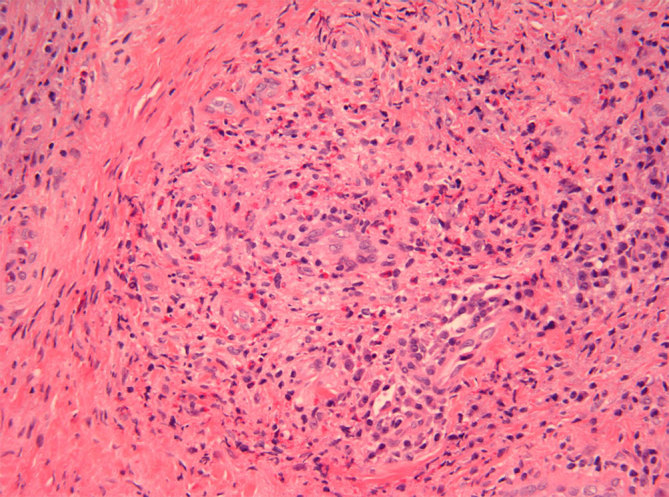
Patient 2. Nasal biopsy demonstrating prominent perivascular fibrosis with numerous eosinophils (haematoxylin and eosin).

On examination, the nasal septum was expanded anteriorly with a firm submucosal mass obstructing an estimated 50% of each nasal airway. MRI showed the anterior septal mass extending into the pre-maxillary space with partial erosion of the hard palate (Figure 3).

**Figure 4.**
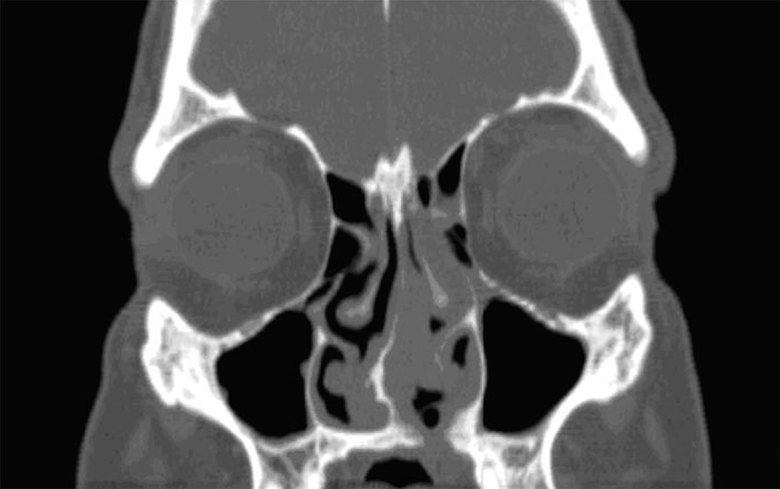
Patient 2. Coronal CT scan showing a nasal mass measuring 2.6 × 3.1 × 2.9 cm predominantly to the left of the nasal septum and extending across the midline to the right. The lesion also extends into the premaxillary space. The erosion through the left hard palate is significant.

Biopsy of the nasal mass revealed extensive perivascular fibrosis in an “onion-skin” pattern, as well as mixed inflammatory infiltrate, including eosinophils, few plasma cells and lymphocytes, consistent with EAF (Figure 4).

Total resection of the mass would have required removal of the caudal septum, anterior nasal spine and areas of hard palate, which would be difficult to reconstruct. Given the minor nature of his symptoms and no significant nasal stenosis or mass deformity, observation was recommended. Subtotal resection will be considered if the patient progresses.

## Discussion

EAF is a benign, inflammatory, fibrosing lesion that was first described by Holmes and Panje^1^ (1983) as a variant of granuloma faciale. Roberts and McCann^2^ (1985) then reported three similar intranasal cases and the detailed histological features, coining the phrase EAF. To date, there have been little more than 45 known reported cases, although there are likely some that have remained undiagnosed.

EAF has a clinical presentation, anatomical predilection and pathognomonic histopathology leading to its diagnosis. Males and females are affected equally, and the average age at diagnosis is 48 years, with a range of 19–72 years.^3^ Symptoms at presentation include nasal obstruction, epistaxis and breathing difficulties.^2,4–9^ More rarely, epiphora and proptosis may be implicated in cases of ocular adnexae involvement.^10^ EAF predominantly affects the upper airways, most commonly presenting in the nasal septum and progressing to the lateral nasal wall.^11^ Thus, EAF should be included in the differential for septal thickening, despite being an uncommon cause.^10^ Around 75% of cases are limited to the nasal septum, lateral nasal wall and sinuses.^3^ There have been fewer case reports of subglottic,^2,4,12^ orbital^10,13–15^ and progressive pharyngeal and soft palate involvement.^16^

The natural history of EAF is that of a benign indolent disease with slow growth, and no malignant transformation has been reported. The slow progression of non-specific symptoms results in an average delay in presentation and diagnosis of 5 years.^3,5,13^ There is some evidence that the lesion stabilizes over time,^3^ as seems to be the case with our first patient. There are no previous reports of fatality, but subglottic and ocular involvement have resulted in significant morbidity.^2,4,12^

Both CT scan and MRI can be non-specific. The typical imaging finding is soft tissue thickening of the septum and lateral nasal walls.^1–20^ Lesions are likely to be longstanding and slowly progressive, with ill-defined margins.^18^ In later stages, adjacent cartilage and bone remodeling, erosion or even sclerosis may be present, although there is generally a lack of bony destruction.^5,18^ On non-enhanced CT scan, EAF may show as an opacification or submucosal mass with homogeneous isodensity compared with grey matter, and calcification is rarely present.^18^ *T*_1_ weighted images generally show EAF lesions to be isointense relative to grey matter, with moderate inhomogeneous enhancement with contrast. On *T*_2_ weighted images, a notable feature is low signal intensity. Lesions are seldom isointense or hyperintense, owing to the fibrosis in late-phase lesions.^18^

The differential diagnosis for EAF is broad, including polyangitis with granulomatous inflammation (formerly known as Wegener’s granulomatosis), Churg–Strauss syndrome, Kimura disease, granuloma faciale, lymphoma, inflammatory pseudotumour, angiolymphoid hyperplasia with eosinophilia, erythema elevatum diutinum and infective granulomatosis.^17,19^ These are excluded based on laboratory investigations and histopathology.^17^

The diagnosis is confirmed with histopathology.^2,5^ EAF progresses from an early inflammatory lesion—with infiltration of eosinophils, plasma cells and lymphocytes—to a late dense fibrous thickening with a perivascular pattern that is comparable with “onion skin”.^2,3^ EAF has a clear histological appearance by which it is diagnosed, but its aetiology remains unknown. Deshpande^21^ (2011) proposed that EAF could be one of the many manifestations of IgG4-related systemic disease after observing that the lesions showed several characterizing features of IgG4-related systemic disease in four of the five patients with EAF.^21^ In addition, trauma and allergy have been suggested as possible aetiologies for EAF.^2,22^

Management of EAF has been challenging, as no definitive treatment exists. Even with the treatment of choice (surgical resection), only about 30% have remained free of recurrence at short-term follow-up.^2–4,10,11,13,20^ Recurrence occurring in 70% of patients is usually at the same anatomic site, requiring multiple resections.^21^ Interestingly, reports of definitive eradication of the lesion have only come from first-time resections.^3^ Thus, given that lesions are slowgrowing and often stabilize over time,^3^ it would be appropriate to avoid radical surgery in the absence of progression and severely disabling symptoms. Other non-surgical physical therapies, including yttrium aluminium garnet and pulsed dye lasers have met with limited long-term success.^7,23^ There is one case of EAF in the literature that was treated with surgery and radiotherapy, but lost to follow-up.^24^ Furthermore, any potential benefits need to be balanced with morbidities associated with radiotherapy for a non-cancerous lesion, including the increased risk of developing malignancy as well as the risks to vision with lesions presenting close to the eye. Medical treatment modalities have also been non-definitive. Only marginal improvement has been noted with local and systemic immunosuppressive therapies; glucocorticoids have been used to control growth, but have not been shown to halt the progression of EAF.^2,5,7,10,13,19^ At times, treatment has had to be discontinued owing to intolerable side effects.^10^ Steroid-sparing agents such as mycophenolate mofetil and azathioprine have been recommended based on limited data. There are also reports of azathioprine, hydroxychloroquine, dapsone, anti-fibrotic agents such as tamoxifen, and antihistamines being used without clear benefit.^3,10^ Further research is needed to establish inhibition of growth in EAF.

## Conclusions

EAF is a rare, benign fibro-inflammatory disease most commonly manifesting as slow-growing but potentially disfiguring lesions of the sinonasal tract. There is currently no definitive treatment. More widespread recognition of this condition will hopefully encourage earlier presentation to specialists and resultant biopsy to help inform management options and improve outcomes.

## Learning points

1. EAF is a benign indolent disease involving the upper airways with slow growth, and no malignant transformation has been reported. Although benign, the potentially disfiguring and obstructing course of this lesion has resulted in significant morbidity with extension into the skeletal muscle and bone, particularly when involving the subglottis and orbit.
2. EAF is most commonly seen in the nasal septum and should be included in the differential for septal thickening. Despite being rare, inflammatory and fibrosing lesions in general should still be considered as part of the differential diagnosis in patients presenting with obstructive symptoms in the sinonasal tract.
3. Although both CT scan and MRI can be non-specific, there are some typical imaging features, which might be helpful in identifying EAF: (a) soft tissue opacification of the septum, lateral nasal walls and sinuses; (b) long-standing and slowly progressive natural history; (c) ill-defined margins; and (d) cartilaginous and bony erosion in late stages, generally with lack of bony destruction.
4. Surgical resection is the treatment of choice, but often results in recurrence. Medical options have been less effective. Also, the risks and morbidity inherent to radiotherapy and medical therapies may outweigh any potential benefit if EAF symptoms remain tolerable and stable.

## Consent

Informed consent was obtained.
